# Postpartum hemorrhage: risk factors for severe blood loss, surgical intervention and peripartum hysterectomy

**DOI:** 10.1007/s00404-025-07969-w

**Published:** 2025-02-11

**Authors:** Emma Barth, Rüdiger Klapdor, Lars Brodowski, Peter Hillemanns, Constantin von Kaisenberg, Vivien Dütemeyer

**Affiliations:** 1 Hannover Medical School (MHH), Gynecology and Obstetrics, Carl-Neuberg-Str. 1, 30625 Hannover, Germany; 2 Albertinen Diakoniewerk, Gynecology and Obstetrics, Hamburg, Germany

**Keywords:** Postpartum hemorrhage, Severe blood loss, Surgical management, Peripartum hysterectomy

## Abstract

**Purpose:**

To evaluate risk factors in patients presenting with postpartum hemorrhage (PPH) associated with severe blood loss (BL), surgical intervention or peripartum hysterectomy.

**Methods:**

This retrospective cohort study included all patients who gave birth at the Hannover Medical School between January 2013 and August 2022 with PPH, defined as BL ≥ 500 ml after vaginal delivery and ≥ 1000 ml after cesarean section. The threshold for severe PPH was set at BL ≥ 1500 ml. Operative management included manual placental removal and/or aspiration/curettage, need for intrauterine balloon tamponade, uterine packing with a chitosan covered gauze or compression sutures. Hysterectomy as ultima ratio was observed separately.

**Results:**

In total 20.9% of 1038 patients with PPH developed severe BL. Several risk factors were identified such as nicotine abuse (aOR 3.45, 95% CI 1.57–7.59, p = 0.002), multiparity (aOR 2.12, 95% CI 1.10–4.10, p = 0.03), uterine malformation (aOR 5.09, 95% CI 1.22–21.16, p = 0.03), c-section (aOR 3.92, 95% CI 2.59–5.92, p < 0.001), placenta praevia (aOR 2.82, 95% CI 1.2–6.63, p = 0.02), abnormal placentation (aOR 9.76, 95% CI 4.22–22.56, p < 0.001) and inversion of the uterus (aOR 16.89, 95% CI 1.62–176.12, p = 0.02). More than one third of the women had a surgical intervention. Independent risk factors for an operative management of PPH were uterus malformation (aOR 5.04, 95% CI 1.22–20.91, p = 0.03), placenta praevia (aOR 2.84, 95% CI 1.23–6.53, p = 0.01), abnormal placentation (aOR 9.78, 95% CI 4.30–22.27, p < 0.001) and c-section (aOR 4.65, 95% CI 3.14–6.89, p < 0.001). Peripartum hysterectomy occurred in 1.9% of the cases and was in addition independently associated wih preeclampsia (aOR 7.50, 95% CI 1.29–43.81, p = 0.03) and amniotic infection syndrome (aOR 12.22, 95% CI 1.92–77.90, p = 0.01).

**Conclusion:**

PPH is a common complication in modern obstetrics and one in five patients with pathological bleeding after birth develops severe BL. There are specific risk factors associated with a BL ≥ 1500 ml, surgical intervention and peripartum hysterectomy in PPH that should be assessed by health professionals and taken into account in the management of this postpartum complication.

**Supplementary Information:**

The online version contains supplementary material available at 10.1007/s00404-025-07969-w.

## What does this study add to the clinical work

**Table:** 

In the case of PPH it is important to be aware of risk factors, such as placentation disorders, uterine malformation, nicotine abuse and multiparity, leading to severe BL causing maternal morbidity in order to escalate therapy, prepare for possible transfusion and multidisciplinary management. 38% of patients require a surgical intervention, which can be organized in a timely manner with knowledge of the associated risk factors.	

## Introduction

Postpartum hemorrhage (PPH) is a leading cause of maternal morbidity and mortality worldwide [1]. The risk of PPH-related complications is higher in low-income countries where access to quality care is limited [2]. But even in high-income countries, where the risk of PPH-related maternal death is rare, the incidence rate continues to rise [3, 4].

This trend has been attributed to an increase in abnormal placentation, labor induction, instrumental delivery and cesarean sections (c-section) [5]. Other risk factors include older maternal age, obesity, preeclampsia, multiple pregnancies, fetal macrosomia, previous PPH, polyhydramnios, antepartum hemorrhage, coagulation disorders and anemia [4–6]. Although there is some evidence exploring risk factors and stratify patients into low, intermediate and high risk groups to prepare prenatally for PPH, the most challenging aspect for clinical practice remains the fact that, in absolute terms, the majority of patients are in the low-risk group. [3, 7–10].

The definition of PPH varies in different guidelines [2, 11–13]. Commonly it is defined as blood loss (BL) ≥ 500 ml within 24 h after birth [14]. The German guideline distinguishes between vaginal birth and c-section, setting the cut-off for the latter at ≥ 1000 ml BL [5]. Others add the presence of signs or symptoms of hypovolemia to the definition of PPH [11, 13, 15]. Normally, up to 1500 ml BL is compensated without hemodynamic complications and symptoms of shock [9, 16, 17]. Beyond that, it can cause severe maternal morbidity including renal or hepatic failure, Sheehan syndrome or respiratory distress syndrome.

Prenatal preparation of resources in the labor ward and the implementation of standardized management algorithms significantly improve the outcome. Nevertheless, the incidence of PPH in western-countries, considered having a high standard of care, is increasing, while most patients with pathologic BL after birth do not present any risk factors [4, 6, 18].

Therefore, the aim of our study was to focus on women with PPH and examine this group for risk factors associated with the development of severe, clinically relevant, bleeding, and the different treatment methods ranging from medical management to surgical intervention to peripartum hysterectomy.

## Methods

### Study participants and design

This retrospective study was conducted at the Hannover Medical School, a tertiary care university hospital in Germany. Included were all patients between January 2013 and August 2022 who had a PPH within 24 h after birth [5]. Exclusion criteria were: incomplete data and postnatal emergency admission for PPH after external birth.

According to the criteria of the German AWMF guideline, PPH was defined as BL ≥ 500 ml after vaginal birth and ≥ 1000 ml after c-section [5]. Due to the fact that PPH with BL ≥ 1500 ml can cause severe hemodynamic complications and symptoms of shock [9, 16, 17], this cut-off was set for the definition of a severe PPH.

The volume of BL was assessed following a standard operating procedure. After visually an excessive blood loss is recognised, the lost blood and continuous blood loss is quantified by weighing the drapes, compresses and blood clots. Most members of the medical staff (midwives, obstetricians, anesthesiologists) undergo an in-house multidisciplinary training in which emergency scenarios including PPH are practiced, called PROMPT (Practical Obstetrics Multi-Professional Training). The training contains lectures and a practical part with visual evaluation of BL and the practice of a simulated PPH emergency in the clinical area [19].

After birth every woman in our unit routinely receives intravenous oxytocin for the placental period, 3 IE after vaginal delivery and 5 IE during c-section. Patients presenting risk factors, usually receive additional prophylactic continuous infusion of oxytocin. An anesthesiologist is available 24 h a day for the maternity ward, which is equipped with its own operating room in the same corridor.

The utilisation of the chitosan-covered gauze was initiated in the labour ward in 2018. Consequently, the analysis of cases pertaining to this surgical intervention was confined to the period subsequent to the aforementioned year.

The study was approved by the local ethics committee (approval number: 10623_BO_K_2022) and the requirement for informed consent was waived.

### Data collection

Patient and delivery characteristics were automatically extracted from the clinical information system. Detailed information, surgical reports and missing data were collected manually from the electronic medical records.

Possible risk factors for PPH were selected after a review of the literature, including established risk factors such as abnormal placentation or previous PPH, as well as controversial risk factors such as nicotine abuse or uterine malformation [6, 20, 21].

### Statistical analyses

Statistical analysis was performed using Microsoft Excel, Microsoft PowerPoint, GraphPad Prism for Windows (version 10.2.1), SPSS software (IBM SPSS version 29.0.1.0). Continuous variables were expressed in median and interquartile range (IR), while categorical variables were summarized as numbers (frequence). Dichotomous potential risk factors were analysed using Chi-square test for unmatched data, whereas Mann–Whitney-U-test and Fishers exact test were used for continuous variables. Univariate logistic analysis was conducted to assess candidate variables as risk factors for PPH, quantified by the odds ratio (OR) and 95% confidence interval (CI). The significant explanatory variables were included in the multivariate logistic analysis. Missing values were not considered. Statistical significance was set at p < 0.05.

## Results

During the study period, 26.004 women gave birth at Hannover Medical School. Of these, 1.074 patients were diagnosed with PPH, representing an incidence rate of 4%. 36 patients were excluded from the study due to incomplete data or postnatal emergency admission for PPH following external birth. In 20.9% (n = 1038) the women developed severe PPH (n = 217). No patient died in the study cohort.

Maternal characteristics for patients with BL ≥ 1500 ml in comparison to those with lower BL are detailed in Table 1. Severe BL was more likely among deliveries with the following characteristics: age > 35 years, nicotine abuse, prepartum anemia, previous uterine surgery, uterus malformation, polyhydramnios, placenta praevia, multiple pregnancy, c-section, abnormal placentation, premature placental abruption, inversion of the uterus and uterus rupture.

**Table 1:** Maternal, pregnancy-associated and delivery-associated characteristics for blood loss 500–1499 ml and ≥ 1500 ml

	Blood loss 500–1499 ml (n = 821)	Blood loss ≥ 1500 ml (n = 217)	aOR	95% CI	p
Maternal characteristics					
Age > 35 years	243 (29.6%)	89 (41.0%)	1.65	1.21–2.25	0.001
Obesity (BMI > 30 in kg/m^2^)	103 (12.5%)	29 (13.4%)	1.08	0.69–1.67	0.75
Nicotine abuse	17 (2.1%)	15 (6.9%)	3.50	1.70–7.16	< 0.001
Anticoagulation medication	57 (6.9%)	8 (3.7%)	0.51	0.24–1.09	0.08
Coagulation disorder	15 (1.8%)	3 (1.4%)	0.75	0.22–2.63	0.66
Prepartum anemia	29 (3.5%)	17 (7.8%)	2.32	1.25–4.31	0.008
Preeclampsia	20 (2.4%)	6 (2.8%)	1.14	0.45–2.87	0.78
Previous uterine surgery	83 (10.1%)	47 (21.7%)	2.46	1.66–3.65	< 0.001
Previous PPH	15 (1.8%)	3 (1.4%)	0.75	0.22–2.63	0.66
Uterus myomatosous	23 (2.8%)	8 (3.7%)	1.33	0.59–3.01	0.50
Uterus malformation	4 (0.5%)	6 (2.8%)	5.81	1.62–20.77	0.007
Pregnancy-associated characteristics					
Parity					
0	504 (61.4%)	107 (49.3%)	0.61	0.45–0.83	0.001
1–2	287 (35.0%)	84 (38.7%)	1.18	0.86–1.60	0.31
≥ 3	30 (3.7%)	26 (12.0%)	3.59	2.07–6.21	< 0.001
Multiples	35 (4.3%)	27 (12.4%)	3.19	1.89–5.40	< 0.001
Polyhydramnios	15 (1.8%)	10 (4.6%)	2.60	1.15–5.86	0.02
Placenta praevia	10 (1.2%)	32 (14.7%)	14.03	6.76–29.05	< 0.001
Abnormal placentation	9 (1.1%)	34 (15.7%)	16.76	7.9–35.56	< 0.001
Induced labour	326 (39.7%)	56 (25.8%)	0.53	0.38–0.74	< 0.001
Gestational age	279 (270–285)	270 (253–282)	0.98	0.98–0.99	< 0.001
Delivery-associated characteristics					
Oxytocin	365 (44.5%)	89 (41.0%)	0.87	0.64–1.18	0.36
Tocolysis	58 (7.1%)	23 (10.6%)	1.57	0.94–2.61	0.08
PROM	307 (37.4%)	71 (32.7%)	0.83	0.60–1.14	0.24
Duration of birth stages					
Latent stage of labour (in h)	4.33 (2.83–6.50)	4.17 (2.40–6.83)	0.98	0.92–1.04	0.55
Second stage of labour (in h)	0.70 (0.33–1.50)	0.9 (0.26–1.69)	1.19	1.00–1.43	0.06
Time of active pressing (in h)	0.13 (0.08–0.20)	0.17 (0.08–0.25)	1.51	0.44–5.14	0.51
Period of time between birth and placenta > 60 min	123 (15.0%)	37 (17.1%)	1.17	0.78–1.74	0.45
Mode of delivery					
Vaginal delivery	733 (89.3%)	121 (55.8%)	0.15	0.11–0.21	< 0.001
c-section	88 (10.7%)	96 (44.2%)	6.61	4.67–9.35	< 0.001
Primary caesarian delivery	49 (6.0%)	60 (27.6%)	6.02	3.98–9.11	< 0.001
Secondary caesarian delivery	23 (2.8%)	33 (15.2%)	6.22	3.57–10.85	< 0.001
Macrosomia (≥ 95% percentile)	70 (8,5%)	19 (8.8%)	1.04	0.61–1.76	0.9
Perineal tear	43 (5.2%)	12 (5.5%)	1.06	0.55–2.05	0.86
Premature placental abruption	11 (1.3%)	14 (6.5%)	5.08	2.27–11.35	< 0.001
Inversion of the uterus	1 (0.1%)	4 (1.8%)	15.40	1.71–138.49	0.02
Uterus rupture	0 (0.0%)	6 (2.8%)	–	–	–
Sepsis	2 (0.2%)	3 (1.4%)	5.74	0.95–34.57	0.06
AIS	7 (0.9%)	4 (1.8%)	2.18	0.63–7.53	0.22

Univariate logistic regression, presented as n (%), adjusted odds ratio (aOR) and 95% confidence intervals (CI)

Abnormal placentation including placenta accreta, increta and percreta

*AIS* amniotic infection syndrome, *BMI* body mass index, c-section cesarean section, *PPH* Postpartum hemorrhage *PROM* premature rupture of membranes

**Fig. 1:**
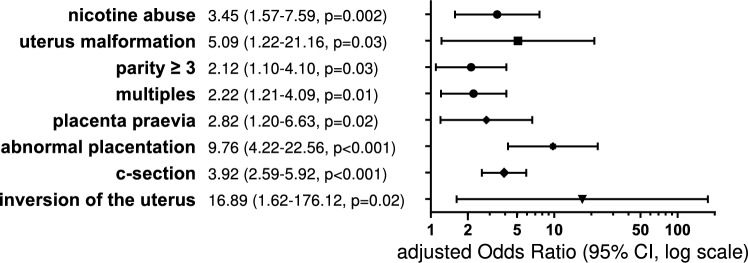
Forest plots for multivariate logistic regression for blood loss ≥ 1500 ml. Abnormal placentation including placenta accreta, increta and percreta. *AIS* amniotic infection syndrome, *CI* confidence interval, *c-section* cesarean section

Multiple logistic regression determined risk factors associated with severe blood loss in the case of PPH (Fig. 1). Nicotine abuse had an adjusted odds ratio of 3.45 (95% CI 1.57–7.59, p = 0.002) and the fact that the mother had her third or more birth was also significantly associated with BL ≥ 1500 ml (aOR 2.12, 95% CI 1.10–4.10, p = 0.03). The presence of a uterine malformation was found to be a significant factor in the increased risk of blood loss ≥ 1500 ml (aOR 5.09, 95% CI 1.22–21.16, p = 0.03). Delivery by c-section had a higher risk for severe BL (aOR 3.92, 95% CI 2.59–5.92, p < 0.001). No differences were seen between primary or secondary c-section. Placental factors were significantly associated with a BL ≥ 1500 ml, such as placenta praevia (aOR 2.82, 95% CI 1.2–6.63, p = 0.02) and abnormal placentation (aOR 9.76, 95% CI 4.22–22.56, p < 0.001). Inversion of the uterus as an obstetrics emergency increased the risk of a bleeding greater than 1500 ml (aOR 16.89, 95% CI 1.62–176.12, p = 0.02).

We examined the risk factors associated with the need for surgical intervention. Intervention includes at least one of the following procedures: manual placental removal and/or aspiration/curettage, need of a intrauterine balloon tamponade, uterine compression sutures and application of a chitosan covered gauze.

**Fig. 2:**
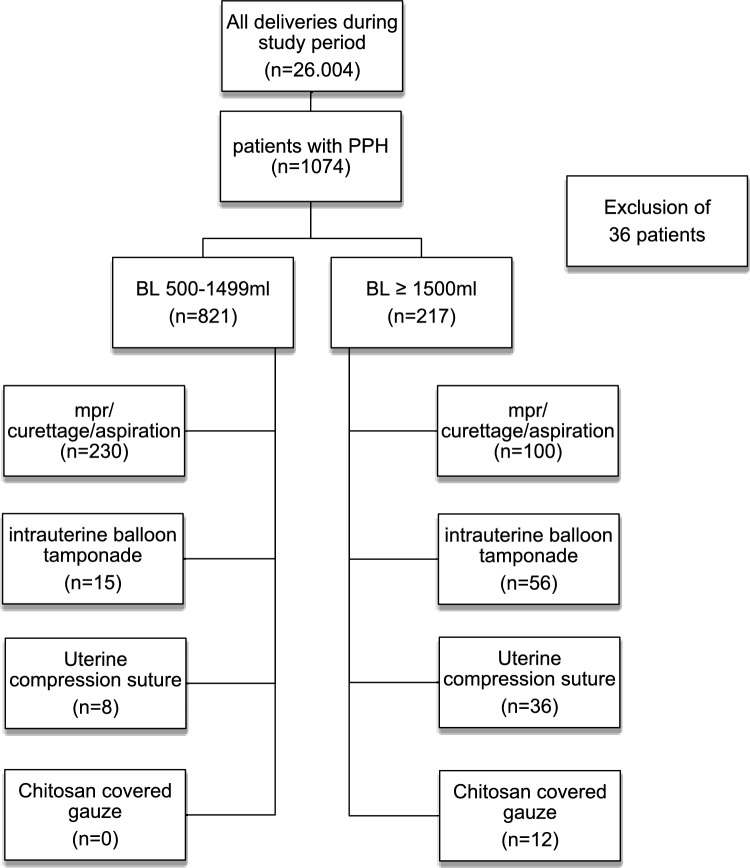
Cohort design for surgical intervention. *mpr* manual placental removal. *BL* blood loss, *PPH* postpartum hemorrhage

Overall 38.1% patients needed an operative intervention. Of those, 38.5% patients had BL ≥ 1500 ml. Manual placental removal and/or aspiration/curettage was the most frequent used method and performed in 28% of patients with BL < 1500 ml and 46.1% with severe BL. Whereas other surgical interventions were performed in the majority of cases in the group with ≥ 1500 ml BL: intrauterine balloon tamponade (1.8% vs. 25.8%), uterine compression sutures (1.6% vs. 16.6%) and chitosan covered gauze (0% vs. 5.5%) (Fig. 2).

**Fig. 3:**
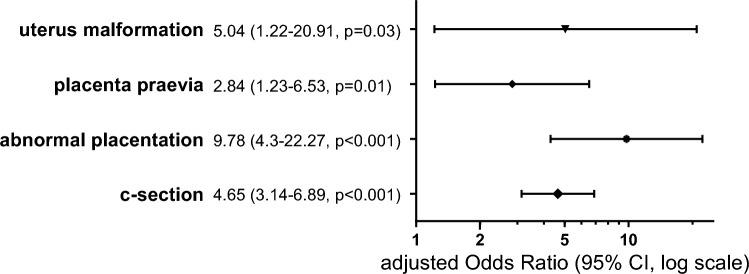
Forest plots for multivariate logistic regression for surgical intervention. Abnormal placentation including placenta accreta, increta and percreta. *CI* confidence interval, *c-section* cesarean section

Risk factors associated with a surgical intervention are presented in the supplementary Table 1. The following variables were independently associated with an operative management: uterus malformation (aOR 5.04, 95% CI 1.22–20.91, p = 0.03), placenta praevia (aOR 2.84, 95% CI 1.23–6.53, p = 0.01), abnormal placentation (aOR 9.78, 95% CI 4.30–22.27, p < 0.001) and c-section (aOR 4.65, 95% CI 3.14–6.89, p < 0.001) (Fig. 3).

**Fig. 4:**
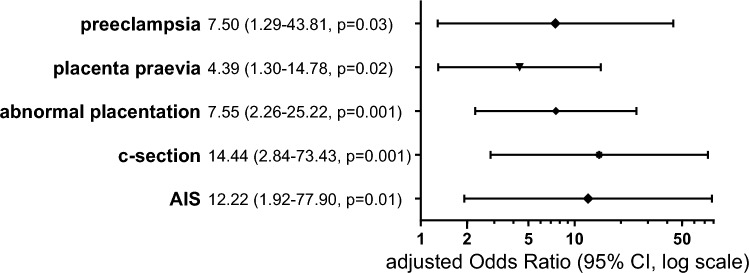
Forest plots for multivariate logistic regression for peripartum hysterectomy. Abnormal placentation including placenta accreta, increta and percreta. *AIS* amniotic infection syndrome, *CI* confidence interval, *c-section* cesarean section

In the study period peripartum hysterectomy occurred in 20 cases, with an incidence of 1.9%. Baseline characteristics are detailed in Supplementary Table 2. A multiple logistic regression determined that the risk for hysterectomy after delivery due to PPH was significantly higher in women with preeclampsia (aOR 7.50, 95% CI 1.29–43.81, p = 0.03), placenta praevia (aOR 4.39, 95% CI 1.30–14.78, p = 0.02), abnormal placentation (aOR 7.55, 95% CI 2.26–25.22, p = 0.001), c-section (aOR 14.44, 95% CI 2.84–73.43, p = 0.001) and amniotic infection syndrome (aOR 12.22, 95% CI 1.92–77.90, p = 0.01) (Fig. 4).

## Discussion

One in five patients with pathological bleeding after birth develop severe BL even in a tertiary hospital in an industrialized country. There are specific risk factors associated with BL ≥ 1500 ml, surgical intervention and peripartum hysterectomy, in the case of PPH. Operative management is common regardless of the severity of PPH, whereas peripartum hysterectomy is rarely performed.

The incidence of PPH in our study of 4% was comparable to others reported in Germany, Ireland, USA or Canada, which ranged from 3 to 8% [15, 18, 22, 23]. Evidence suggests that the rate is gradually increasing. This phenomenon was also observed in the present cohort, with initial incidences of 4% and a subsequent rise to 4.5% over a period of nearly ten years [4, 23]. It is imperative to acknowledge that the population of patients at risk in our university center is higher compared to smaller centers. This may explain why the rate of PPH is not lower despite the available treatment resources and trained medical staff [20].

Patients can be stratified prenatally into risk groups for PPH using the California Maternal Quality Care Collaborative Assessment Tool [24]. Prenatal pretransfusion testing is recommended according to the classification. However, these prediction models are controversial, as it has been demonstrated that 43% of PPH occurs in the low-risk group and that they have a low predictive value for the development of severe bleeding in the high-risk group [10]. Furthermore, the course of birth is not considered, despite the fact that it comprises the greatest risk factors, such as prolonged labour or the placental period, secondary caesarean sections for the development of PPH, or, as observed in the present study, the appearance of severe blood loss [25]. Thus, Dilla et al. suggest a pretransfusion type and screening only in high-risk patients, and state clearly that there should be a case-by-case assessment including risk factors that develop as the delivery progresses [10].

As a tertiary center in an industrialized country with standardized protocols, regular training for emergencies including PPH and the anytime accessibility of operative management, the rate of surgical intervention in total and especially in the group of BL < 1500 ml might be higher compared to centers having a more difficult access to these escalated procedures. The WHO acknowledged an inequality in healthcare between countries worldwide [26]. Even within the same developing country the quality of care varies between urban and rural areas. Low- and middle-income countries have limited opportunities to implement international algorithms as there is reduced availability of resources and expertise [27]. But also, in centers across Europe there is a considerable variation of peripartum hysterectomy, performed in the majority of cases for uterine atony and abnormally invasive placenta [28]. This illustrates that the recommendation to establish standardized and adequate management of PPH or in particular placenta accreta spectrum should be followed with amendments based on local characteristics and available resources [27, 29, 30].

Identification of patients with abnormal placentation or position of the placenta and preparation of at-risk patients for delivery is crucial. Placenta accreta spectrum as well as placenta praevia have the highest likelihood of ending up with a PPH[5, 21]. Despite knowing these important risk factors, most of these patients in our cohort developed severe bleeding. Thus, severe cases are difficult to prevent but an adequate prenatal preparation may reduce morbidity and mortality [31]. In clinical practice it is in addition of great interest for the medical staff to be aware of risk factors in patients presenting with PPH leading to severe, hemodynamically relevant and causing morbidity and mortality, blood loss and to the likelihood of surgical intervention. Especially in centers where operative management requires time for organization, this could be of beneficial value.

While some studies have found an association between obesity and the development of PPH, others have not [22, 32]. A meta-analysis from 2021 was also unable to identify obesity to be a risk factor, assuming that probably underlying conditions such as diabetes mellitus or hypertension are the real risk factors, leaving the clinical impression that obesity increases PPH [21]. We did not see more obese women presenting severe BL in PPH-patients. It could be assumed that medical treatment fails more often in mothers with a higher BMI, as research suggested a decrease in uterine contractility and as the dose for oxytocin for an adequate uterine response seems to be higher [33–35]. However, we did not observe neither more severe BL nor a greater risk for surgical treatment, which is in line with the findings of Polic et al. who did not observe any difference between obese and non-obese women regarding the rate of intrauterine pressure balloon tamponade or peripartum hysterectomy [36]. As a consequence, in obese patients the treatment protocol should be maintained until all conservative measures have been exhausted.

In our study, nicotine was found to be a risk factor for progression to severe BL in the PPH group. Few studies did not observe a significant association between smoking and PPH [20, 37, 38]. However, the recent meta-analysis by Huang et al. showed that nicotine abuse is rarely evaluated [20, 37, 38]. It is suggested that nicotine induces hepatic cytochrome P450 (CYP) isoenzymes and as a consequence effect the metabolism of some medications [39]. Whether nicotine affects the bioavailability of oxytocin, sulproston or misoprostol in the medical treatment of PPH, resulting in higher doses needed to reduce severe BL, needs to be investigated. Sulproston is a prostaglandin E2 derivative (PGE2). A small sample size study analysing the human placenta saw that nicotine prevented the effect of PGE2, in releasing endothelium-derived relaxing factor, a vasodilator of placental arteries [40]. Again, whether this pathomechanism plays a role in patients who smoke and experience PPH has to be the subject of further research.

C-section is an established risk factor for PPH [6, 41, 42]. We observed more cases of severe BL in this group. Furthermore, there was a higher probability of further surgical intervention, such as the use of intrauterine balloon tamponade or gauze packing. C-section significantly increased the possibillity of peripartum hysterectomy. In 18 of 20 cases of peripartum hysterectomy a c-section was performed. It might be possible that the inhibition threshold of the medical staff to use operative management is lower in patients who underwent operative abdominal delivery compared to patients who have had vaginal birth.

The most common surgical procedure is the removal of the placenta or aspiration/curettage. It is evident that the most significant risk factors pertain to placental complications, including but not limited to a protracted placental period, placental pathologies, and low gestational age, which are characterised by an increased propensity for placental tissue retention [43, 44]. In the case of persistent atony, other methods follow [45, 46]. This is reflected in our observations and recommended in the German guideline for the management of PPH [5].

There is need to standardize the definition of peripartum hysterectomy in the context of PPH to improve the comparability of national surveys [28]. The rate in our cohort was low at 1,9% compared to 5,7% reported in a study of cases managed at a tertiary center using the same definition for PPH [36]. Similar rates were reported in a study from a university hospital in Oslo for patients with severe BL ≥ 1500 ml (1,6% vs. 2.1% in our study) [47]. We also identified placental conditions as the well known greatest risk factor for hysterectomy especially in high-income countries [28, 47–49]. Interestingly, we observed significantly more cases of preeclampsia and amniotic fluid infection syndrome in the group who underwent peripartum hysterectomy. It is imperative that clinicians take this into consideration.

The study has some limitations. First, the retrospective design. Second, the assessment of BL was initially visually following a quantitative method for the accurate measurement of blood loss when PPH was detected. The visual estimation of blood loss is known to be less accurate compared to the gravimetric technique [50]. It is more likely to underestimate the actual blood loss when volumes are high and overestimate when volumes are low [51]. Therefore, it can be supposed that every PPH-case was detected.The discrimination of severity was warranted with the quantitative method. The regular training of the medical staff with the multidisciplinary training program PROMPT is supposed to improve the visual estimation of blood loss [52]. It was shown, that PROMPT in fact ameliorate neonatal outcome but does not reduce cases with PPH [53, 54]. Whether it has an effect on the development of severe PPH or PPH-related maternal morbidity should be the subject of further research. However, there is no evidence that accurate measurement of BL improves outcome and that one method is superior to the other [55–57]. It has been suggested that BL volume plays a minor role in the detection of clinically important PPH and that the nature and speed of postpartum bleeding as well as the condition of the mother are crucial in the decision-making process of management [55].

## Conclusion

PPH is a common complication in modern obstetrics. Prenatal risk assesment is important to identify high risk cases and make preparations, but in the event of PPH, risk factors should be reassessed and a professional multidisciplinary team should be prepared for potential conventional and surgical interventions. Further prospective research is needed to validate the risk factors identified.

## Supplementary Information

Below is the link to the electronic supplementary material.

Supplementary file1 (DOCX 27 KB)

## Data Availability

No datasets were generated or analysed during the current study.
