# Supplementary material for: Postpartum hemorrhage: risk factors for severe blood loss, surgical intervention and peripartum hysterectomy
Source: Arch Gynecol Obstet. 2025 Feb 11;312(1):167–76. doi: 10.1007/s00404-025-07969-w (PMC12177015; doi:10.1007/s00404-025-07969-w)
Supplement: Supplementary file 1 — Supplementary file1 (DOCX 27 KB) [file 404_2025_7969_MOESM1_ESM.docx]

**SUPPLEMENTARY**

**Supp. Table 1:** Maternal, pregnancy-associated and delivery-associated characteristics for surgical intervention

|  | no surgical intervention n = 643 | surgical intervention n = 395 | aOR | 95% CI | p |
| --- | --- | --- | --- | --- | --- |
| **Maternal characteristics** |  |  |  |  |  |
| age > 35 years | 181 (28.1%) | 151 (38.2%) | 1.58 | 1.21-2.06 | < 0.001 |
| obesity (BMI > 30 in kg/m^2^ ) | 76 (11.8%) | 56 (14.2%) | 1.23 | 0.85-1.79 | 0.27 |
| nicotine abuse | 18 (2.8%) | 14 (3.5%) | 1.28 | 0.63-2.60 | 0.50 |
| anticoagulation medication | 43 (6.7%) | 22 (5.6%) | 0.82 | 0.49-1.40 | 0.47 |
| coagulation disorder | 10 (1.6%) | 8 (2.0%) | 1.31 | 0.51-3.34 | 0.57 |
| prepartum anemia | 25 (3.9%) | 21 (5.3%) | 1.39 | 0.77-2.52 | 0.28 |
| preeclampsia | 16 (2.5%) | 10 (2.5%) | 1.02 | 0.46-2.27 | 0.97 |
| previous uterine surgery | 69 (10.7%) | 61 (15.4%) | 1.52 | 1.05-2.20 | 0.03 |
| previous PPH | 11 (1.7%) | 7 (1.8%) | 1.04 | 0.40-2.70 | 0.94 |
| uterus myomatosous | 20 (3.1%) | 11 (2.8%) | 0.89 | 0.42-1.88 | 0.77 |
| uterus malformation | 2 (0.3%) | 8 (2.0%) | 6.63 | 1.40-31.36 | 0.02 |
| **Pregnancy-associated characteristics** |  |  |  |  |  |
| parity |  |  |  |  |  |
| 0 | 395 (61.4%) | 216 (54.7%) | 0.76 | 0.59-0.98 | 0.03 |
| 1-2 | 220 (34.2%) | 151 (38.2%) | 1.19 | 0.92-1.54 | 0.19 |
| ≥ 3 | 28 (4.4%) | 28 (7.1%) | 1.68 | 0.98-2.87 | 0.06 |
| multiples | 32 (5.0%) | 30 (7.6%) | 1.57 | 0.94-2.63 | 0.09 |
| polyhydramnios | 11 (1.7%) | 14 (3.5%) | 2.11 | 0.95-4.70 | 0.07 |
| placenta praevia | 16 (2.5%) | 26 (6.6%) | 2.76 | 1.46-5.22 | 0.002 |
| abnormal placentation | 8 (1.2%) | 35 (8.9%) | 7.72 | 3.54-16.82 | < 0.001 |
| induced labour | 250 (38.9%) | 132 (33.4%) | 0.79 | 0.61-1.03 | 0.08 |
| gestational age (in days) | 279 (270-285) | 275 (261-283) | 0.98 | 0.98-0.99 | < 0.001 |
| **Delivery-associated characteristics** |  |  |  |  |  |
| oxytocin | 289 (44.9%) | 165 (41.8%) | 0.88 | 0.68-1.13 | 0.32 |
| tocolysis | 41 (6.4%) | 40 (10.1%) | 1.66 | 1.05-2.62 | 0.03 |
| PROM | 235 (36.5%) | 143 (36.2%) | 1.003 | 0.77-1.30 | 0.98 |
| Duration of birth stages |  |  |  |  |  |
| latent stage of labour (in h) | 4.5 (3.0-6.8) | 4.0 (2.33-6.33) | 0.96 | 0.92-1.003 | 0.07 |
| second stage of labour (in h) | 0.73 (0.33-1.48) | 0.70 (0.25-1.67) | 1.06 | 0.92-1.22 | 0.43 |
| time of active pressing (in h) | 0.13 (0.08-0.20) | 0.13 (0.08-0.21) | 0.89 | 0.34-2.34 | 0.81 |
| period of time between birth and placenta > 60 min | 11 (1.7%) | 149 (37.7%) | 34.80 | 18.54-65.33 | < 0.001 |
| mode of delivery |  |  |  |  |  |
| vaginal delivery | 549 (85.4%) | 305 (77.2%) | 0.58 | 0.42-0.80 | <0.001 |
| c-section | 94 (14.6%) | 90 (22.8%) | 1.72 | 1.25-2.38 | <0.001 |
| primary caesarian delivery | 56 (8.7%) | 55 (13.9%) | 1.69 | 1.14-2.51 | 0.01 |
| secondary caesarian delivery | 28 (4.4%) | 26 (6.6%) | 1.55 | 0.89-2.68 | 0.12 |
| macrosomia | 59 (9.2%) | 30 (7.6%) | 0.82 | 0.52-1.29 | 0.38 |
| perineal tear | 37 (5.8%) | 18 (4.6%) | 0.78 | 0.44-1.39 | 0.40 |
| premature placental abruption | 18 (2.8%) | 7 (1.8%) | 0.63 | 0.26-1.51 | 0.30 |
| inversion of the uterus | 1 (0.2%) | 4 (1.0%) | 6.57 | 0.73-58.97 | 0.09 |
| uterus rupture | 3 (0.5%) | 3 (0.8%) | 1.63 | 0.33-8.13 | 0.55 |
| sepsis | 3 (0.5%) | 2 (0.5%) | 1.09 | 0.18-6.53 | 0.93 |
| AIS | 3 (0.5%) | 8 (2.0%) | 4.41 | 1.16-16.72 | 0.03 |

Univariate logistic regression, presented as n (%), adjusted odds ratio (aOR) and 95% confidence intervals (CI)

*abnormal placentation* including placenta accreta, increta and percreta *AIS* amniotic infection syndrome *BMI* body mass index *c-section* caesarean section *PPH* postpartum haemorrhage *PROM* premature rupture of membranes

**Supp. Table 2:** Maternal, pregnancy-associated and delivery-associated characteristics for peripartal hysterectomy

|  | no hysterectomy necessary n = 1018 | hysterectomy necessary n = 20 | aOR | 95% CI | p |
| --- | --- | --- | --- | --- | --- |
| **Maternal characteristics** |  |  |  |  |  |
| age > 35 years | 323 (31.7%) | 9 (45.0%) | 1.76 | 0.72-4.29 | 0.21 |
| obesity (BMI > 30 in kg/m^2^ ) | 128 (12.6%) | 4 (20.0%) | 1.74 | 0.57-5.28 | 0.33 |
| nicotine abuse | 31 (3.0%) | 1 (5.0%) | 1.68 | 0.22-12.92 | 0.62 |
| anticoagulation medication | 65 (6.4%) | 0 (0.0%) | -- | -- | 0.00 |
| coagulation disorder | 17 (1.7%) | 1 (5.0%) | 3.10 | 0.39-24.49 | 0.28 |
| prepartum anemia | 42 (4.1%) | 4 (20.0%) | 5.81 | 1.86-18.14 | 0.002 |
| preeclampsia | 24 (2.4%) | 2 (10.0%) | 4.60 | 1.01-20.96 | 0.05 |
| previous uterine surgery | 122 (12.0%) | 8 (40.0%) | 4.90 | 1.96-12.22 | < 0.001 |
| previous PPH | 18 (1.8%) | 0 (0.0%) | -- | -- | 0.00 |
| uterus myomatosous | 30 (2.9%) | 1 (5.0%) | 1.73 | 0.23-13.38 | 0.60 |
| uterus malformation | 10 (1.0%) | 0 (0.0%) | -- | -- | 0.00 |
| **Pregnancy-associated characteristics** |  |  |  |  |  |
| parity |  |  |  |  |  |
| 0 | 607 (59.6%) | 4 (20.0%) | 0.17 | 0.06-0.51 | 0.002 |
| 1-2 | 360 (35.4%) | 11 (55.0%) | 2.23 | 0.92-5.44 | 0.08 |
| ≥ 3 | 51 (5.0%) | 5 (25.0%) | 6.32 | 2.21-18.07 | < 0.001 |
| multiples | 59 (5.8%) | 3 (15.0%) | 2.87 | 0.82-10.06 | 0.1 |
| polyhydramnios | 22 (2.2%) | 3 (15.0%) | 7.99 | 2.18-29.26 | 0.002 |
| placenta praevia | 32 (3.1%) | 10 (50.0%) | 30.81 | 11.98-79.24 | < 0.001 |
| abnormal placentation | 34 (3.3%) | 9 (45.0%) | 23.68 | 9.20-60.92 | < 0.001 |
| induced labour | 379 (37.2%) | 3 (15.0%) | 0.30 | 0.09-1.02 | 0.05 |
| gestational age (in days) | 278 (268-284) | 254 (241-262) | 0.98 | 0.97-0.99 | < 0.001 |
| **Delivery-associated characteristics** |  |  |  |  |  |
| oxytocin | 450 (44.2%) | 4 (20.0%) | 0.32 | 0.11-0.95 | 0.04 |
| tocolysis | 77 (7.6%) | 4 (20.0%) | 3.05 | 1.00-9.35 | 0.05 |
| PROM | 373 (36.6%) | 5 (25.0%) | 0.61 | 0.22-1.72 | 0.35 |
| duration of birth stages |  |  |  |  |  |
| latent stage of labour (in h) | 4.25 (2.83-6.63) | 3.08 (2.12-3.08^1^) | 0.71 | 0.34-1.51 | 0.38 |
| second stage of labour (in h) | 0.73 (0.32-1.50) | 0.14 (0.10-0.14^1^) | -- | -- | 0.20 |
| time of active pressing (in h) | 0.13 (0.08-0.20) | 0.05 (0.03-0.05^1^) | -- | -- | 0.11 |
| period of time between birth and placenta > 60min | 159 (15.6%) | 1 (5.0%) | 0.28 | 0.04-2.14 | 0.22 |
| mode of delivery |  |  |  |  |  |
| vaginal delivery | 852 (83.7%) | 2 (10.0%) | 0.02 | 0.01-0.09 | <0.001 |
| c-section | 166 (16.3%) | 18 (90.0%) | 46.19 | 10.62-200.96 | <0.001 |
| primary caesarian delivery | 97 (9.5%) | 12 (60.0%) | 14.24 | 5.68-35.69 | < 0.001 |
| secondary caesarian delivery | 50 (4.9%) | 6 (30.0%) | 8.30 | 3.06-22.50 | < 0.001 |
| macrosomia | 89 (8.7%) | 0 (0.0%) | -- | -- | 0.00 |
| perineal tear | 55 (5.4%) | 0 (0.0%) | -- | -- | 0.00 |
| premature placental abruption | 24 (2.4%) | 1 (5.0%) | 2.18 | 0.28-16.95 | 0.46 |
| inversion of the uterus | 5 (0.5%) | 0 (0.0%) | -- | -- | 0.00 |
| uterus rupture | 6 (0.6%) | 0 (0.0%) | -- | -- | 0.00 |
| sepsis | 4 (0.4%) | 1 (5.0%) | 13.34 | 1.42-125.05 | 0.02 |
| AIS | 9 (0.9%) | 2 (10.0%) | 12.46 | 2.51-61.79 | 0.002 |

Univariate logistic regression, presented as n (%), adjusted odds ratio (aOR) and 95% confidence intervals (CI)

*abnormal placentation* including placenta accreta, increta and percreta *AIS* amniotic infection syndrome *BMI* body mass index *c-section* caesarean section *PPH* postpartum haemorrhage *PROM* premature rupture of membranes

s
